# Antimicrobial Peptides and Interleukins in Cleft Soft Palate

**DOI:** 10.3390/children10071162

**Published:** 2023-07-02

**Authors:** Arina Deņisova, Māra Pilmane, Dzintra Kažoka

**Affiliations:** Institute of Anatomy and Anthropology, Riga Stradins University, Kronvalda Boulevard 9, LV-1010 Riga, Latvia; mara.pilmane@rsu.lv (M.P.); dzintra.kazoka@rsu.lv (D.K.)

**Keywords:** cleft palate, interleukin 10, human beta defensins, cathelicidin

## Abstract

Cleft palate is one of the most common and well-studied congenital anomalies; however, the role of protective tissue factors in its pathophysiology is still debated. The aim of our study was to evaluate interleukin and antimicrobial peptide appearance and distribution in cleft palate. Eight soft palate samples were obtained during veloplasty procedures. Immunohistochemical staining was applied to detect HBD-2-, HBD-3-, HBD-4-, LL-37-, IL-10-, and CD-163-positive cells via light microscopy. For statistical evaluation, the Mann–Whitney U test and Spearman’s rank correlation coefficient were used. A significant difference between study groups was observed for HBD-2 and IL-10 in epithelial and connective tissue as well as HBD-4 in connective tissue. The number of HBD-3-positive cells was moderate in the patients, and few were observed in the controls. The number of LL-37-positive cells varied from a moderate amount to a numerous amount in both study groups, whilst CD-163 marked a moderate number of positive cells in patients, and a few-to-moderate amount was observed in the controls. Numerous correlations between studied factors were revealed in cleft tissues. The increase in antimicrobial peptides HBD-2 and HBD-4 and anti-inflammatory cytokine IL-10 suggested a wide compensatory elevation of the local immune system against cleft-raised tissue changes. The correlations between the studied factors (HBD-2, HBD-3, HBD-4, LL-37, and IL-10) proved the synergistic involvement of common local defense factors in postnatal cleft palate morphopathogenesis.

## 1. Introduction

Cleft lips and/or palates (CLPs) are common congenital malformations affecting the oral cavity and its related structures. In Europe, the prevalence of CLPs is as high as 1.55 live births per 1000, with the most prevalent variant being the combined cleft lip and palate, whereas isolated cleft palates occur in around 1 out of every 2000 births [1,2]. Since CLPs are linked to a variety of breathing and feeding issues in the early postpartum period, as well as dental and psychosocial problems, all of which have an impact on the patient’s quality of life, it is important to make sure that the patient receives a proper treatment, including family counseling, surgical interventions, and psychological and social support [3,4,5].

The etiology of CLPs is multifactorial. Approximately 30% of cleft lip and/or palate cases and half of isolated cleft palate cases are associated with malformation syndromes, some of which have identified mutated genes; for example, the IRF6 gene in Van der Woude syndrome and the SOX9 gene in Pierre Robin syndrome [6,7]. Non-syndromic clefts also have a genetic basis, such as variants in WNT genes, yet most of the cases are sporadic [6,8,9]. Environmental factors involved in the pathogenesis of CLPs include maternal smoking, alcohol use, and advanced age of parents [10,11,12]. Although both genetics and environmental factors have been widely studied, knowledge regarding the morphopathogenesis, especially regarding protective tissue factor involvement in cleft palate, is limited.

Previous studies indicated that human beta defensins 2, 3, and 4 (HBD-2, -3, and -4) as well as human cathelicidine (LL-37), interleukin 10 (IL-10), and hemoglobin scavenger receptor CD-163 were all found in the oral cavity (and, in some cases, saliva) due to different conditions, yet previous research on the presence of these factors in the soft palate, and especially on patients affected by clefts, is scarce [13,14,15,16,17,18,19]. A study conducted by Casaroto et al. showed that palate epithelial primary cells can produce HBD-2 at a baseline level as well as in the case of *Candida albicans* exposure, and IL-10 was proven by Seidel et al. to be increased in orofacial cleft patient tongue smears, yet there is still a lack of studies on these factors in cleft-affected soft palate tissues, specifically [19,20]. Thus, this study was oriented toward local defense factors in cleft palate patients that could take part and have an important role in postnatal local tissue protection.

HBD-2, a member of the beta defensin family, is a strong antimicrobial peptide that is secreted by epithelial cells throughout an organism in response to different pro-inflammatory signals and microbial antigens. Its appearance has been noted in the respiratory, gastric, and intestinal tract mucosa; gingival epithelium; salivary glands; saliva; and skin, where it supports host defense mechanisms [13,14,21,22,23,24]. Moreover, HBD-2 and HBD-3 are more pronounced in the oral mucosa than in skin, which shows their importance in the antimicrobial protection of the oral cavity [25]. HBD-2 is also present in the cartilage of patients affected with cleft lip and palate, suggesting that there could be an inflammatory reaction in these tissues, which could possibly influence wound healing afterwards [26].

Antimicrobial protein HBD-3 is expressed in the stomach, lungs, and skin in response to inflammatory cues and pathogens [27,28,29]. HBD-3 induces the inflammatory signaling, chemotaxis, and degranulation of mast cells; the production of prostaglandins; and dendritic cell activation and migration [30,31,32]. Moreover, HBD-3 promotes wound healing via angiogenesis and fibroblast activation [33]. HBD-3 is also expressed in the oral mucosa epithelial cells (for example, in case of exposure to cigarette smoke), and it has been proven to be protective against periodontitis development in mice models [15,34,35].

HBD-4 is a potent antibacterial agent found in organs such as the lungs, stomach, testis, and uterus, as well as in dental pulp, where it is induced by pro-inflammatory cytokines TNFα and IL-1α. One of its functions is to downregulate the expression of IL-1α, IL-1β, IL-6, and TNFα [36,37,38,39]. HBD-4 differs from other defensins with selective, salt-sensitive antimicrobial activity and chemotaxis, mostly for monocytes, which supports the idea that all beta defensins have their own role in providing immunity [37]. For example, regarding COVID-19, HBD-4 was shown to be increased in the sera of positive patients, whilst HBD-2 was decreased [40]. In the oral mucosa, HBD-4, similarly to other defensins, is also expressed in healthy gingival epithelia and those affected by chronic periodontitis [16].

LL-37 is another important defense molecule that is released by neutrophils, macrophages, keratinocytes upon stimulation by TNFα, lipopolysaccharides (LPSs), and the activation of Toll-like receptors (TLRs) [41,42]. LL-37 causes the direct perforation of plasma membranes, enhances phagocytosis by macrophages, and induces pro-inflammatory molecule synthesis [43,44,45]. LL-37 has proangiogenic properties. It improves wound re-epithelization and induces fibroblast proliferation and migration, yet its pro-proliferative properties also have a role in lung cancer [46,47,48,49]. LL-37 is expressed in human saliva and the gingival epithelium, where it inhibits bacterial growth and biofilm formation and protects from caries [17,50,51].

IL-10 is a pleiotropic cytokine produced by B lymphocytes and macrophages as well as epithelial cells in, for example, the intestines [52,53,54]. IL-10 inhibits the activity of other cytokines such as IL-12, TNFα, and IFNα and supports cell differentiation into antibody-secreting cells, which proves that IL-10 is a powerful regulator of immune processes [55,56]. IL-10 has also been studied in oral health; it is expressed in the oral mucosa in relation to acute wounds [57]. Furthermore, IL-10 has been proven to be present in cleft patients’ nasal septum cartilage and cleft lip tissues, where it takes part in the stimulation of cell proliferation and in providing a balance in the immune response [58,59].

Lastly, CD-163 is a monocyte- and macrophage-specific hemoglobin scavenger receptor that is regulated by a variety of pro- and anti-inflammatory factors. It is commonly used as an M2 macrophage group marker; it significantly increases upon the presence of IL-6 and IL-10 and is downregulated by TNFα and IFN-γ [60,61]. Regarding immunity, CD-163 activation has an effect via the secretion of anti-inflammatory cytokines and the activity of heme metabolites, which altogether suppress inflammation [62]. Interestingly, CD-163 has been also noted in M2 macrophages in healthy and cleft-affected lips, where it possibly protects the tissues from excessive inflammation [63].

Since the role of protective tissue factors in the morphopathogenesis of cleft malformations is still debated, the goal of our study was to determine the presence and distribution of local defense factors—anti-inflammatory interleukins and antimicrobial peptides—in cleft-affected palate tissues.

## 2. Materials and Methods

### 2.1. Material Characteristics of Subjects

The study was conducted in accordance with the Helsinki Declaration in Latvia at Riga Stradins University’s Institute of Anatomy and Anthropology. This research was approved by the Research Ethics Committee at Riga Stradins University, and a permit was issued in May 2003 and December 2022 (May 22, 2003; December 14, 2022; Nr. 2-PEK-4/595/2022). Eight soft palate tissue samples were obtained from children diagnosed with cleft palate and/or lip aged 6 to 12 years during veloplasty procedures (Table 1). The inclusion criteria were mixed dentition age, diagnosis of cleft palate, and indication for plastic surgery, whereas the exclusion criteria were genetic and chromosomal abnormalities and immunodeficiency. The control soft palate material was obtained from two male and three female subjects aged 40 to 60 years without orofacial clefts or any inflammatory signs. The control group tissue material was gathered from the Institute of Anatomy and Anthropology of Riga Stradins University and consisted of post-mortem necropsies. The study and control groups were considered comparable because they belong to the mixed dentition age, which is the period after the milk dentition age.

### 2.2. Routine Morphological Assessment

Fixation of the obtained tissue material was performed using 2% formaldehyde, 0.2% picric acid, and 0.1 M phosphate buffer with a pH of 7.2 for 24 h. The samples were then processed for 12 h in Tyrode’s buffer containing 10% saccharose. Subsequently, the tissues were embedded in paraffin and cut with a microtome into 5 μm sections. Hematoxylin and eosin staining was performed to evaluate the morphological structure of the soft palate.

### 2.3. Immunohistochemical (IHC) Analysis

The biotin—streptavidin method was used for the immunohistochemical labeling and detection of HBD-2 (sc-20798, working dilution 1:100, Santa Cruz Biotechnology Inc., Dallas, TX, USA), HBD-3 (orb183268, working dilution 1:100, Biorbyt LLC, St Louis, MO, USA), HBD-4 (ab70215, working dilution 1:100, Abcam, Cambridge, UK), LL-37 (orb88370, working dilution 1:100, Biorbyt LLC, St Louis, MO, USA), IL-10 (orb100193, working dilution 1:600, Biorbyt LLC, St Louis, MO, USA), and CD-163 (ab87099, working dilutions 1:200, Abcam, Cambridge, UK) [64,65].

Antibody diluent (code-938B-05, Cell Marque^TM^, Rocklin, CA, USA) was used to dilute all antibodies in this study. The obtained soft palate tissue cuts were deparaffinized and washed in alcohol and water, rinsed two times for 5 min in TRIS buffer solution (code-2017X12508, Diapath S.p.A., Martinengo, Italy), and then were placed for 20 min in a microwave with boiling EDTA buffer (code-2017X02239, Diapath S.p.A., Martinengo, Italy). The cooled tissues were washed twice for 5 min with TRIS buffer, blocked with 3% peroxide solution for 10 min, and washed again with TRIS buffer. Tissue samples were then incubated with primary antibodies for 1 h and washed three times with TRIS buffer. Next, a HiDef Detection^TM^ reaction amplificator (code 954D-31, Cell Marque^TM^, Rocklin, CA, USA) was used for 10 min at room temperature. Tissues were washed again with TRIS buffer and then incubated for 10 min at room temperature with a HiDef Detection^TM^ HRP Polymer Detector (code-954D-32, Cell Marque^TM^, Rocklin, CA, USA). The last wash with TRIS buffer was applied three times, each for 5 min. Tissue coating was performed for 10 min with a DAB+ chromogenic liquid DAB Substrate Kit (code 957D-60, Cell Marque**^TM^**, Rocklin, CA, USA) after which the samples were rinsed with running water and counterstained with hematoxylin (code-05-M06002, Mayer’s Hematoxylin, Bio Optica Milano S.p.A., Milano, Italy). Finally, the tissues were dehydrated with increasing concentrations (from 70° to 90°) of ethanol, clarified with carboxylic acid and xylol, and sealed with a coverslip.

Light microscopy and semi-quantitative non-parametric analysis were used to evaluate immunoreactive cell appearance and distribution. Positively stained cells in the epithelium and connective tissue of the soft palate in the visual field were graded using a scale containing the following labels: 0—no positive cells; 0/+—occasional positive cells; +—few positive cells; +/++—few to moderate positive cells; ++—moderate positive cells; ++/+++—moderate to numerous positive cells; +++—numerous positive cells [66].

### 2.4. Statistical Analysis

Data analysis was performed following the evidence-based statistical analysis and methods in biomedical research (SAMBR) checklist [67]. As the positive cell count was graded on a scale instead of using their exact quantity, non-parametric tests were used to calculate the results. The Mann–Whitney U test was performed to detect statistically significant differences in the studied factors between patient and control samples. Spearman’s rank correlation coefficient was used for the evaluation of correlations between the cytokines and antimicrobial peptides, in which R < 0.2 was assumed as a very weak correlation, R = 0.20–0.39 indicated a weak correlation, R = 0.40–0.59 indicated a moderate correlation, R = 0.60–0.79 indicated a strong correlation, and R = 0.80–1.00 indicated a very strong correlation. All results with a *p*-value ≤ 0.05 were considered statistically significant. Data processing was performed using IBM SPSS software version 27.0 (IBM company, North Castle, Armonk, NY, USA).

## 3. Results

### 3.1. Routine Changes

**Figure 1:**
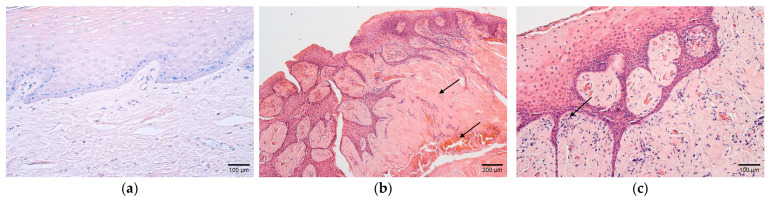
(**a**–**c**) Histological view of the soft palate structures in controls and children with cleft palate. (**a**) Control specimen containing unchanged stratified squamous non-keratinized epithelium and mucosal connective tissue. Hematoxylin and eosin, ×200. (**b**) Connective tissue abundance and rich vascularity in the cleft soft palate (arrows). Hematoxylin and eosin, ×100. (**c**) Note the inflammatory infiltrate in the subepithelial connective tissue in a patient sample (arrow). Hematoxylin and eosin, ×200.

The collected and examined tissue samples of the soft palate included non-keratinized stratified squamous epithelium and mucosal connective tissue (Figure 1a). The hematoxylin and eosin staining of cleft palates revealed rich vascularity, thickening of the connective tissue, and inflammatory cell infiltration (Figure 1b,c).

### 3.2. Immunohistochemistry

**Figure 2:**
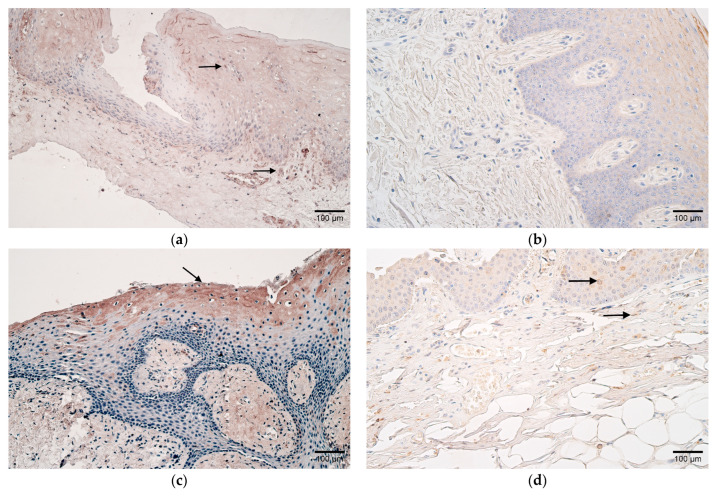

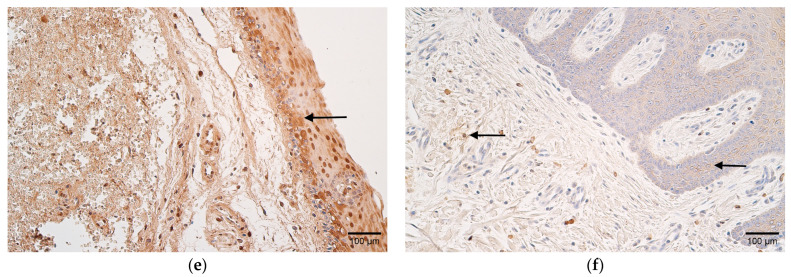
(**a**–**f**) Histological view of the soft palate in children with cleft palate and controls. (**a**) Few HBD-2-positive cells in the epithelium and moderate positive cells in the subepithelial connective tissue of a patient (arrows). HBD-2 IMH, ×200. (**b**) Absence of HBD-2-positive cells in the connective tissue of a control sample. HBD-2 IMH, ×200. (**c**) Note patient sample containing moderate HBD-3-positive epitheliocytes (arrow). HBD-3 IMH, ×200. (**d**) Few HBD-3-positive cells in the epithelium and connective tissue of control (arrows). HBD-3 IMH, ×200. (**e**) Patient sample containing moderate HBD-4-positive epitheliocytes (arrow). HBD-4 IMH, ×200. (**f**) Note a few HBD-4-positive epithelial and connective tissue cells in a control sample (arrows). HBD-4 IMH, ×200.

In contrast to the control samples, which contained only occasional HBD-2-positive cells in the epithelium and none in the connective tissue, few HBD-2-positive cells were discovered in the epithelium, and a moderate number were found in the connective tissues of the patients (Table 2 and Table 3, Figure 2a,b).

The number of HBD-3-positive cells in patients varied from none to numerous in the epithelium and connective tissue, yet controls contained mostly few positive cells in both (Table 2 and Table 3, Figure 2c,d).

Interestingly, HBD-4 also revealed diversity in patients with positive cells that ranged from few to numerous in the epithelium and connective tissues, whereas control samples primarily had few (Table 2 and Table 3, Figure 2e,f).

**Figure 3:**
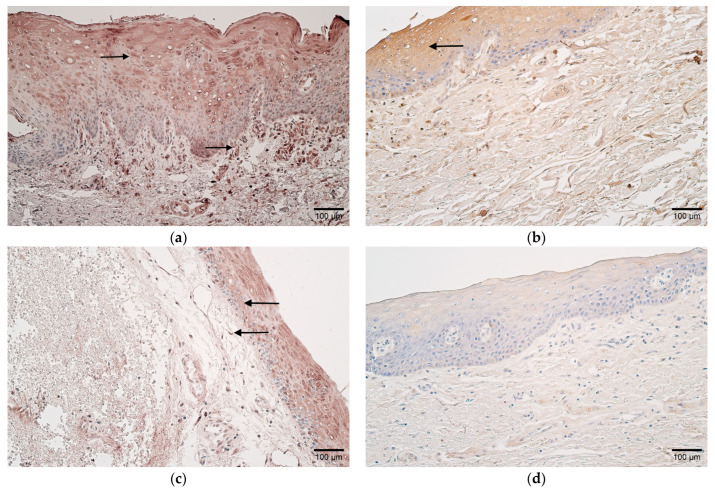
(**a**–**d**) Histological view of the soft palate in children with cleft palate and controls. (**a**) Numerous LL-37-positive cells in the epithelium and connective tissue of a patient (arrows). LL-37 IMH, ×200. (**b**) Moderate number of LL-37-positive cells in the epithelium of a control (arrow). LL-37 IMH, ×200. (**c**) Patient sample containing moderate-to-numerous IL-10-positive epithelial cells and moderate positive cells in connective tissue (arrows). IL-10 IMH, ×200. (**d**) Note the absence of IL-10-positive cells in the connective tissue of a control sample. IL-10 IMH, ×200.

A moderate-to-numerous number of LL-37-positive cells were marked in the epithelium and connective tissue of the patients. Similarly, the controls contained mostly moderate numbers of positive cells in both (Table 2 and Table 3, Figure 3a,b).

In the epithelium of patients, a moderate-to-numerous number of IL-10-positive cells were noted, but in connective tissues, they varied from occasional to numerous. Few epithelial and occasional connective tissue IL-10-positive cells were observed in the controls (Table 2 and Table 3, Figure 3c,d).

**Figure 4:**
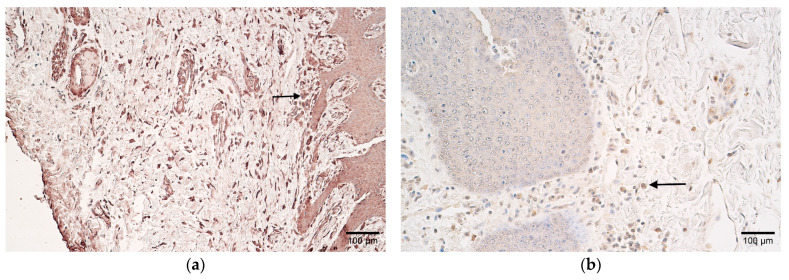
(**a**,**b**) Histological view of the soft palate in children with cleft palate and controls. (**a**) Moderate number of CD-163-positive macrophages in patient tissues (arrow). CD-163 IMH, ×200. (**b**) Control sample containing few-to-moderate CD-163-positive macrophages (arrow). CD-163 IMH, ×200.

CD-163 marked a moderate number of positive macrophages in the patient samples, and few to a moderate number in the controls (Table 2 and Table 3, Figure 4a,b).

Statistically significant differences using the Mann–Whitney U test between the patient and control samples were noted in IL-10- and HBD-2-containing epithelial cells as well as IL-10-, HBD-2-, and HBD-4-positive cells of connective tissues (Table 4).

Spearman’s rank correlation coefficient showed a very strong positive correlation in the cleft-affected soft palate epithelium between HBD-2 and IL-10 and HBD-3 and HBD-4. There was also a very strong correlation noted between HBD-3 and HBD-4, HBD-3 and IL-10, and HBD-4 and IL-10 in the connective tissue. Interestingly, epithelial HBD-3 and HBD-4 correlated very strongly with connective tissue HBD-3, HBD-4, and IL-10. Lastly, a strong positive correlation was also noted in the connective tissue between HBD-2 and HBD-4 as well as LL-37 and IL-10 (Table 5).

## 4. Discussion

Although the pathogenesis of cleft palate has been extensively studied, the function of the immunological mechanisms in this process is still being investigated. The relationship between cleft pathologies and different complications, such as dysbiosis, postoperative wound healing disorders, and gingival inflammation, further supports the hypothesis that immunity also contributes to the condition [68,69]. One of the most important and first-line defense mechanisms of the oral mucosa is the epithelium, which covers the soft palate and provides a barrier that protects against mechanical or chemical stress and pathogen invasion. Moreover, the underlying connective tissue or lamina propria further supports the epithelium by providing mechanical support, allowing the diffusion of nutrients and other molecules to the epithelium, and by participating in inflammatory reactions as it also contains immune cells [70].

In our study, statistically significant differences were noted in HBD-2- and IL-10-positive cells in the epithelium and connective tissue as well as in HBD-4, which was more prominent only in connective tissue. This means that HBD-2 and IL-10 are the main protectors of the epithelium, but HBD-4 is also the main protector of the connective tissue in cleft-affected palates. Our data are also indirectly supported by the results of other scientists, who found that HBD-2 is a defensin expressed in the oral mucosa, and its appearance has been noted in salivary glands and gingival epithelia in response to various stimuli, including bacterial LPSs and cytokines such as IL-1β [13,14]. HBD-2 has many functions, including the induction of pro-inflammatory cytokine synthesis and recruitment of immunocompetent cells [21]. For example, palate epithelial primary cells produce HBD-2 and nitric oxide (NO) upon direct contact with *Candida albicans*, which supports the evidence that the palate epithelium plays a role in innate immunity [20]. Moreover, in addition to pro-inflammatory properties, HBD-2 has also shown pro-proliferative characteristics on human skin keratinocytes together with a positive effect on their migration, which shows that HBD-2 can be beneficial in wound healing [24]. Furthermore, HBD-2 has different effects on fibroblast activity in connective tissue. A study conducted in 2022 by Umehara et al. showed that HBD-2 induces angiogenin secretion via dermal fibroblasts, supporting angiogenesis, which is particularly beneficial in chronic wounds [71]. HBD-2 also induces human conjunctival fibroblast proliferation, gene expression, and the synthesis of fibroblast collagenase (MMP1), which is required for extracellular matrix remodulation and supporting cell migration during wound healing [72]. Thus, in soft palate clefts, HBD-2 could exhibit more than one function in both the epithelium and connective tissue.

Given that there was a significant increase in IL-10-positive epitheliocytes and connective tissue cells in patients in our study, we suspect that this factor could also be involved in suppressing excessive local inflammation in cleft-affected soft palate tissues. Indeed, IL-10, a cytokine produced by both immune and epithelial cells, is an important regulator of both innate and adaptive immunity [52,53,54]. Its effects have been associated with the suppression of various cytokines, such as IL-1α, IL-1β, IL-6, IL-8, TNFα, and IFNα, as well as the downregulation of class II MHC antigens on monocytes and the stimulation of B cell differentiation into plasmoblasts [55,56,73,74]. IL-10 has been previously identified in the oral cavity, specifically in periodontal ligament cells and in gingival crevicular fluid, and decreased IL-10 levels are associated with the pathogenesis of periodontitis [75,76]. Moreover, IL-10 has been shown to inhibit IL-6 production by human gingival fibroblasts exposed to bacterial LPS, supporting the idea that this factor decreases inflammation in connective tissues in periodontal disease [77]. Another study by Seidel et al. showed that IL-10, together with different pro-inflammatory cytokines such as IL-1β, IL-2, IL-6, and IL-8, appears to be increased in the tongue smears of orofacial cleft-affected neonates, and a more pronounced secretion of these cytokines has been noted in patients with higher orofacial cleft clinical severity [19].

Lastly, HBD-4 was the only defensin that showed a statistically significant difference only in the connective tissue of the cleft soft palate, where it seems to play the most important role. HBD-4 has been previously noted in the bronchial, bronchiolar, gastric, and gingival epithelium as well as in dental pulp stem cells upon pro-inflammatory cytokine stimulation, which results in the lowered production of a variety of inflammatory factors, such as IL-1α, IL-1β, IL-6, and TNF-α [16,37,38,39,78]. Furthermore, HBD-4 has also been proven to induce the heightened mRNA production of specific cytokines and chemokines, such as IL-10, monocyte chemoattractant protein-1, and macrophage inflammatory protein-3α, as well as to increase keratinocyte migration and proliferation [24]. As for connective tissue cells, the HBD-4 gene is expressed by dermal fibroblasts, and it induces angiogenin production with them, promoting angiogenesis, which is required for effective wound repair [71,79]. Thus, similarly to HBD-2, we suggest that HBD-4 may be involved not only in the inflammatory process of cleft-affected tissues but also in wound healing. However, due to the differences in its activity from other defensins, such as its very selective and salt-sensitive antimicrobial properties, the absence of binding sites for NF-κB and STAT in promoter regions that are characteristic of other defensins, and selectivity in monocyte chemotaxis, the functions of HBD-4 may differ from those of HBD-2 [37].

Different correlations between the examined cytokines and antimicrobial agents were observed in this study. First, HBD-3 and HBD-4 showed a very strong positive correlation in both the epithelium and connective tissue, as well as between the epithelium and connective tissue of patients with cleft soft palates. The synergistic activity of both defensins has been previously demonstrated as they can induce similar cytokine production, such as the production of IL-18 and IL-31 [80,81]. Moreover, a study by Harder et al. showed that HBD-3 and HBD-4 are produced by keratinocytes in the presence of proinflammatory cytokines such as TNFα, IL-1β, and IFN-γ, and this process is inhibited by retinoic acid. Thus, inductor signals that increase the expression of these defensins could also overlap, resulting in higher levels of them. This could also explain the strong positive correlation between connective tissue HBD-4 and HBD-2 as HBD-2 production has been previously shown to be associated with the same cytokines [82].

Connective tissue and epithelial HBD-3 and HBD-4 correlated very strongly with IL-10 in the connective tissues of the patient samples, and HBD-2 showed a very strong positive correlation with IL-10 in the epithelium. These correlations could be explained by the fact that all of the mentioned beta defensins (HBD-2, -3, and -4) have been proven to increase IL-10 expression in experimental models, thus further supporting the idea that beta defensins have a multifactorial role in immunity [24,35,83].

LL-37 also strongly correlated with IL-10 in connective tissue, which could be because LL-37, similarly to beta defensins, can induce IL-10 production, as shown in a study by Torres-Juarez et al. in 2015, in which macrophages were proven to synthesize IL-10 in cases of infection with Mycobacterium tuberculosis in the presence of LL-37 [84]. The synergistic activity between antimicrobial peptides and IL-10 supports the idea that both innate and adaptive immune processes could be involved in cleft soft palate tissues.

In our study, CD-163, a marker of M2 macrophage activation, did not reveal statistically significant differences between the study groups. However, factor-positive macrophages were moderate in patients and few-to-moderate in controls. CD-163-positive macrophages have been noted previously in normal tissues, such as those of bone marrow, alveoli of the lungs, liver, skin, and intestines, as well as in different diseases, including colorectal and oral squamous cell cancers [18,85,86]. M2 macrophages are not only important for antigen presentation but have also been described to possess anti-inflammatory properties, such as deactivating pro-inflammatory macrophages, suppressing T lymphocyte proliferation and function, and secreting anti-inflammatory cytokines, such as IL-10 [87,88]. This finding supports the notion that CD-163-positive macrophages may be involved in the provision and regulation of local immunity in both healthy and cleft-affected soft palate tissues.

The factors studied in our research have shown clinical significance in previous studies. For example, HBD-2 can serve as a marker for multiple disease severities, including atopic dermatitis and psoriasis, while IL-10 has been proven to increase together with the severity of orofacial clefts [19,89,90]. Some defense molecules are also used to evaluate the efficacy of treatment. HBD-4 can be used to assess the success of osteoarthritic knee cartilage treatment with autologous cell transplantation [91]. Thus, these factors may also be useful for the prognosis and treatment of cleft palates. The assessment of other factors, for example, anti-inflammatory cytokines, such as IL-4 and IL-13, as well as some pro-inflammatory cytokines like IFN-γ and TNFα, which have been linked to other orofacial cleft tissues, could possibly provide additional information about the inflammatory mechanisms in cleft palate and reveal some correlations with our results [26,92]. Lastly, we suspect that our results would not significantly differ from results concerning the adult population as the children in this study already belonged to the mixed dentition stage and had permanent teeth, which is also characteristic of adults. However, research on the factors studied in children before and during milk dentition may reveal new insights into this topic.

Our study has some limitations; for example, a relatively small number of both patient and control samples could possibly affect the statistical significance and correlations calculated, yet obtaining these tissues is difficult due to ethical considerations. Moreover, immunohistochemistry was the method of choice to evaluate the presence of the studied factors in tissue samples; however, other standardized laboratory tests, such as ELISA, could reveal additional information about tissue factor concentration levels. Furthermore, a semi-quantitative method of positive-cell grading was used; thus, the collected data were in ordinal form, and non-parametric tests were used for the statistical calculations. This method is partially susceptible to subjectivity; however, the results were evaluated by two independent researchers, and it is internationally recognized and widely used in histology as it serves as a good tool for the evaluation of factors in tissues [93,94]. It would also be valuable to identify the genes involved in the pathogenesis of cleft soft palates.

## 5. Conclusions

The increase in cells that are positive for the antimicrobial proteins HBD-2 and HBD-4 and the anti-inflammatory cytokine IL-10 implies that these factors are engaged in the widespread activation of the local immune system to compensate for the alterations in the soft palate tissues caused by clefts. The presence of CD-163-positive macrophages further maintains the idea of the role of local immunity in patients with cleft palates.

The observed correlations between the studied factors (HBD-2, HBD-3, HBD-4, LL-37, and IL-10) in the cleft-affected epithelium and connective tissue prove that they are mutually involved via a synergistic manner in the morphopathogenesis of postnatal cleft palate and that both adaptive and innate immune reactions could be involved in it.

## Figures and Tables

**Table 1:** Characteristics of cleft palate patients of mixed dentition age.

Patient Number	Gender	Age (in Years)	Clinical Diagnosis
1	M	6	Uranoschisis
2	M	6.3	Cheilognathouranoschisis sinistra
3	M	7.1	Uranoschisis partialis
4	F	7.3	Cheilognathouranoschisis sinistra
5	F	8.7	Uranoschisis
6	M	11.1	Cheilognathouranoschisis bilateralis
7	M	11.3	Uranoschisis partialis
8	F	12.7	Cheilognathouranoschisis sinistra

Abbreviations: M—Male; F—female; uranoschisis—cleft palate; cheilognathouranoschisis—cleft lip, alveolar ridge, and palate; partialis—partial; sinistra—left; bilateralis—bilateral.

**Table 2:** Relative number of antimicrobial-peptide-positive and interleukin-positive cells in the patient cleft soft palates.

Patient Number	HBD-2		HBD-3		HBD-4		LL-37		IL-10		CD-163
	E	CT	E	CT	E	CT	E	CT	E	CT	M
1	++	++	++	++	++/+++	+++	+++	++	+++	++	++
2	+	++	++/+++	++/+++	+++	+++	+++	+++	++/+++	+++	+++
3	++	+++	+++	+++	+++	+++	++/+++	+++	+++	+++	+++
4	N	++	N	++	N	++	N	+++	N	++	++/+++
5	N	+/++	N	+/++	N	+/++	N	++	N	+	+/++
6	+	++	0	0	+	+/++	++	++	++/+++	0/+	+
7	+	+/++	+	0	+	+	++/+++	++	++/+++	+/++	+
8	+/++	++	+/++	+/++	+/++	++	++	+++	++/+++	++	+/++
Median value	+	++	++	++	++	++	++/+++	++/+++	++/+++	++	++

Abbreviations: HBD-2—Human beta defensin 2; HBD-3—human beta defensin 3; HBD-4—human beta defensin 4; LL-37—human cathelicidine; IL-10—interleukin 10; CD-163—hemoglobin scavenger receptor; E—epithelium; CT—connective tissue; M—macrophages; N—no epithelium; 0—no positive cells; 0/+—occasional positive cells; +—few positive cells; +/++—few-to-moderate positive cells; ++—moderate number of positive cells; ++/+++—moderate-to-numerous positive cells; +++—numerous positive cells in the visual field.

**Table 3:** Relative number of antimicrobial-peptide-positive and interleukin-positive cells in the control soft palate.

Control Number	HBD-2		HBD-3		HBD-4		LL-37		IL-10		CD-163
	E	CT	E	CT	E	CT	E	CT	E	CT	M
1	0	0/+	+	+	+	+	++	++	+	+	+
2	0/+	0	+	+	+	+	++/+++	+++	+	0/+	+/++
3	0	0/+	+	0/+	0	0/+	++	++	+	+	+/++
4	+	0	+	0/+	+	+	++	++	+	0	++
5	0/+	0	+	+	+/++	+/++	++	++	+	0	+/++
Median value	0/+	0	+	+	+	+	++	++	+	0/+	+/++

Abbreviations: HBD-2—human beta defensin 2; HBD-3—human beta defensin 3; HBD-4—human beta defensin 4; LL-37—human cathelicidine; IL-10—interleukin 10; CD-163—hemoglobin scavenger receptor; E—epithelium; CT—connective tissue; M—macrophages; 0—no positive cells; 0/+—occasional positive cells; +—few positive cells; +/++—few-to-moderate positive cells; ++—moderate number of positive cells; ++/+++—moderate-to-numerous positive cells; +++—numerous positive cells in the visual field.

**Table 4:** Significant differences in interleukins and antimicrobial peptides in the soft palate epithelium and connective tissue between patient and control tissue samples.

Marker	HBD-2 E	HBD-2 CT	HBD-4 CT	IL-10 E	IL-10 CT
Mann–Whitney U	1.500	0.000	3.500	0.000	3.500
*p*-value	0.009	0.002	0.011	0.004	0.011

Abbreviations: IL-10—Interleukin 10; HBD-2—human beta defensin 2; HBD-4—human beta defensin 4; E—epithelium; CT—connective tissue.

**Table 5:** Spearman’s rank correlation coefficient revealing correlations between antimicrobial peptides and interleukins in connective tissue and epithelium of patient samples.

Factor 1	Factor 2	R	*p*-Value
**Very Strong Positive Correlation (R = 0.80–1.00)**			
HBD-2 E	IL-10 E	0.894	0.016
HBD-3 E	HBD-3 CT	0.986	<0.001
HBD-3 E	HBD-4 E	0.971	0.001
HBD-3 E	HBD-4 CT	0.880	0.021
HBD-3 E	IL-10 CT	0.971	0.001
HBD-3 CT	HBD-4 CT	0.906	0.002
HBD-3 CT	IL-10 CT	0.900	0.002
HBD-4 E	HBD-3 CT	0.985	<0.001
HBD-4 E	HBD-4 CT	0.938	0.006
HBD-4 E	IL-10 CT	0.970	0.001
HBD-4 CT	IL-10 CT	0.854	0.007
**Strong Positive Correlation (R = 0.60–0.79)**			
HBD-2 CT	HBD-4 CT	0.763	0.028
LL-37 CT	IL-10 CT	0.788	0.020

Abbreviations: R—Spearman’s rank correlation coefficient; IL-10—interleukin 10; HBD-2—human beta defensin 2; HBD-3—human beta defensin 3; HBD-4—human beta defensin 4; LL-37—human cathelicidine; E—epithelium; CT—connective tissue.

## Data Availability

All datasets used/analyzed in the present study are presented in the Results section of the manuscript.
